# Diagnostic model constructed by nine inflammation-related genes for diagnosing ischemic stroke and reflecting the condition of immune-related cells

**DOI:** 10.3389/fimmu.2022.1046966

**Published:** 2022-12-13

**Authors:** Peng Ren, Jing-Ya Wang, Hong-Lei Chen, Xiao-Wan Lin, Yong-Qi Zhao, Wen-Zhi Guo, Zhi-Rui Zeng, Yun-Feng Li

**Affiliations:** ^1^ Beijing Institute of Basic Medical Sciences, Beijing, China; ^2^ Department of Anesthesiology, Seventh Medical Center of Chinese PLA General Hospital, Beijing, China; ^3^ Department of Anesthesiology, Beijing Shijitan Hospital, Capital Medical University, Beijing, China; ^4^ Guizhou Provincial Key Laboratory of Pathogenesis & Drug Research on Common Chronic Diseases, Department of Physiology, School of Basic Medical Sciences, Guizhou Medical University, Guizhou, China; ^5^ Beijing Institute of Pharmacology and Toxicology, State Key Laboratory of Toxicology and Medical Countermeasures, Beijing Key Laboratory of Neuropsychopharmacology, Beijing, China

**Keywords:** ischemic stroke, inflammation, immunology, diagnostic biomarker, machine learning

## Abstract

**Background:**

Ischemic cerebral infarction is the most common type of stroke with high rates of mortality, disability, and recurrence. However, the known diagnostic biomarkers and therapeutic targets for ischemic stroke (IS) are limited. In the current study, we aimed to identify novel inflammation-related biomarkers for IS using machine learning analysis and to explore their relationship with the levels of immune-related cells in whole blood samples.

**Methods:**

Gene expression profiles of healthy controls and patients with IS were download from the Gene Expression Omnibus. Analysis of differentially expressed genes (DEGs) was performed in healthy controls and patients with IS. Single-sample gene set enrichment analysis was performed to calculate inflammation scores, and weighted gene co-expression network analysis was used to analyze genes in significant modules associated with inflammation scores. Key DEGs in significant modules were then analyzed using LASSO regression analysis for constructing a diagnostic model. The effectiveness and specificity of the diagnostic model was verified in healthy controls and patients with IS and with cerebral hemorrhage (CH) using qRT-PCR. The relationship between diagnostic score and the levels of immune-related cells in whole blood were analyzed using Pearson correlations.

**Results:**

A total of 831 DEGs were identified. Both chronic and acute inflammation scores were higher in patients with IS, while 54 DEGs were also clustered in the gene modules associated with chronic and acute inflammation scores. Among them, a total of 9 genes were selected to construct a diagnostic model. Interestingly, RT-qPCR showed that the diagnostic model had better diagnostic value for IS but not for CH. The levels of lymphocytes were lower in blood of patients with IS, while the levels of monocytes and neutrophils were increased. The diagnostic score of the model was negatively associated with the levels of lymphocytes and positively associated with levels of monocytes and neutrophils.

**Conclusions:**

Taken together, the diagnostic model constructed using the inflammation-related genes *TNFSF10*, *ID1*, *PAQR8*, *OSR2*, *PDK4*, *PEX11B*, *TNIP1*, *FFAR2*, and *JUN* exhibited high and specific diagnostic value for IS and reflected the condition of lymphocytes, monocytes, and neutrophils in the blood. The diagnostic model may contribute to the diagnosis of IS.

## 1 Introduction

Cerebral vascular disease is characterized by acute neurological disease, resulting in a high mortality, disability, and recurrence (1, 2). Among them, ischemic cerebral infarction is the most common type of stroke in clinical settings (3) and a leading cause of long-term disability and death worldwide (4). Currently, the main approved treatment for this type of stroke is revascularization, which has a strict therapeutic window (< 4.5 h) (5). Nevertheless, the vast majority of patients cannot receive thrombolytic therapy upon hospital admission (6, 7). Therefore, earlier and faster identification of acute IS is important, as thrombolytic therapies are time-sensitive (8); blood biomarkers provide a possibility for identification, especially in circumstances where access to brain imaging is limited (9). However, there are currently no blood biomarkers used for the diagnosis of IS due to the required characteristics of high sensitivity and specificity in this heterogeneous disorder and a fast turnaround (10).

As a result of cerebral ischemia, both experimental animal models and patients with stroke experience a strong inflammatory response (11) involving the release of dangerous/damage-associated molecular patterns (DAMPs), highly immunogenic cellular components, from the brain into the systemic circulation (12). Activating these DAMPs causes adaptive and peripheral innate immune cells to migrate to ischemic brain areas, and the inflammatory response in ischemic regions shows a variable positive and negative influence, depending on IS phase, and variable involvement of inflammatory cells (13). A toxic effect is generated when proinflammatory cytokines, proteases, and reactive oxygen species are produced by inflammatory cells. In the penumbra, it may cause secondary damage, causing neuronal death and orchestrating an immune response comprising glial activation and recruitment of peripheral immune cells (14). The protective effects consist of clearance of injured tissue by myeloid cells and the establishment of a regenerative environment. In this way, a large number of researchers have revealed that anti-inflammatory strategies hold great promise in extending the therapeutic window and preventing major brain damage during reperfusion (5, 15). Thus, it is crucial to identify inflammation-associated blood biomarkers in patients with IS that could either enhance the beneficial effects or dampen toxic effects, improving outcome.

In recent years, high-throughput technologies have been rapidly developed, including microarrays and RNA sequencing, and, with their respective data-analysis methods, have provided valuable and effective methods to study the molecular underpinnings of complex diseases (16). For instance, Li et al. (17) reported that SLAMF1, IL-7R, and NCF4 may be novel therapeutic targets to promote functional recovery after IS; Zheng et al. (18) identified four reliable serum markers for the diagnosis of IS and concluded that immune cell infiltration plays a crucial role in the development and progression of IS. Herein, we sought to identify the inflammation-related diagnostic blood biomarkers of patients with IS and their relationship with the levels of immune-related cells in the blood using multi-informatics algorithms to find effective targets for the treatment of IS and lay the groundwork for the development of diagnostic options.

## 2 Materials and methods

### 2.1 Data source and preprocessing

The gene expression profiles in the GSE22255 (including 20 patients with IS and 20 healthy controls) and GSE16561 (including 39 patients with IS and 24 healthy controls) datasets were extracted from the public database Gene Expression Omnibus (GEO; http://www.ncbi.nlm.nih.gov/geo). GSE22255 and GSE16561 were normalized, and differences between batches were removed before defining the integrated gene expression profile. The gene expression profiles were normalized using the limma package. Interbatch differences for the GSE22255 and GSE16561 datasets, including 59 samples from patients with IS and 44 from healthy controls, were eliminated by the ComBat function in the “sva” package in R software.

### 2.2 Identification of DEGs

The limma package in R was used to analyze DEGs between samples from patients with IS and healthy controls. DEGs (|FC| ≥ 1.2 and adjusted *P*-values < 0.05) were identified and plotted in volcano plots and heatmaps.

### 2.3 Determination of inflammation scores in each sample

Seventeen genes of chronic inflammation and 113 genes of acute inflammation were obtained in the gene set “GOBP_CHRONIC_INFLAMMATORY_RESPONSE” and “GOBP_ACUTE_INFLAMMATORY_RESPONSE” from the Gene Set Enrichment Analysis (GSEA; http://www.gsea-msigdb.org/gsea/index.jsp) (**Supplementary Table 1**). A single-sample GSEA (ssGSEA) algorithm was used for calculating the inflammation scores in each sample based on the gene signature. The differences in acute/chronic inflammation scores between the sample groups was determined using an unpaired t test, and *P-values* < 0.05 were considered statistically significant.

### 2.4 Construction of weighted gene co-expression network analysis and identification of modules significantly associated with inflammation

Weighted gene co-expression network analysis (WGCNA) was performed to identify co-expression modules using the R package “WGCNA” (v. 4.0.2). Prior to performing WGCNA, a scale-free network was constructed by removing outlier samples. To further calculate the adjacency values between genes with variance greater than all quartiles of variance, a standard scale-free network was used to approximate the appropriate soft threshold power (β = 6). Then, the adjacency values were transformed into a topological overlap measure (TOM), following which the dissimilarity (1-TOM) values were induced. Finally, modules were obtained using the hierarchical clustering tree algorithm and assigned random colors using 1-TOM dissimilarity. An analysis of Pearson correlations was used to identify modules with biological significance between modules and clinical characteristics. Modules with a |co-relationship| (|R|) ≥ 0.4 and *P* < 0.05 were considered clinically significant. Further analysis was conducted on genes in clinically significant modules with module membership (MM) ≥ 0.6 and gene significance (GS) ≥ 0.05.

### 2.5 Enrichment analysis of interesting modules

Kyoto Encyclopedia of Genes and Genomes and Gene Ontology enrichment analysis of the genes were submitted to the online database of Enrichr (http://amp.pharm.mssm.edu/Enrichr/) to conduct functional and pathway enrichment analysis. The cut-off for significance was set at *P* < 0.05.

### 2.6 Construction of the diagnostic model for IS

Least absolute shrinkage and selection operator (LASSO) algorithms were used to identify the key genes with the best diagnostic value for IS (19). A LASSO logistic regression analysis was performed using the “glmnet” package, and the response type was binomial (α = 1). To minimize bias, we selected the fittest λ and deleted some genes that partially exhibited collinearity. After multivariate logistic regression analysis of the influences generated by the LASSO regression, we selected the relevant parameters with p-values less than 0.05 as the final parameters of the diagnostic model. We calculated risk scores by multiplying each inflammation-associated gene expression level by a linear combination of the corresponding tolerance limits. Finally, to evaluate the diagnostic performance of this model, we used R software and the “pROC” package to determine the area under the curve (AUC) of each receiver operating characteristic (ROC).

### 2.7 Collection of whole blood samples

Participants were recruited from the Seventh Medical Center of the Chinese PLA General Hospital. All patients with IS or CH underwent detailed and rigorous neurological examination. The diagnostic criteria for IS are based on the International Classification of Diseases (9th Revision), and patients with IS are classified into different subtypes according to the modified TOAST classification. Patients with a history of blood disorders, type 1 diabetes, autoimmune, thyroid, tumor, kidney or liver disease are excluded. Finally, whole blood specimens (samples to be discarded after remaining clinical examination) from 15 healthy individuals, 34 cases of IS, and 16 patients with CH were collected and stored at -80°C for further analysis. The study procedures were developed based on the 2008 revision of the Declaration of Helsinki of 1975 (http://www.wma.net/en/30publications/10policies/b3/) and approved by the Ethics Committee of Seventh Medical Center of the Chinese PLA General Hospital (No: 2022-182).

### 2.8 RT-qPCR

RNAprep Pure High Efficiency Total RNA Extraction Kit (Cat no. DP443, TianGen, Beijing, China) was used to extract total RNA in blood serum. Briefly, 800 ng total RNA of each sample was used to perform reverse transcription to synthesis the first chain cDNA using TAKARA PrimeScript RT reagent Kit (Cat no. RR037, TAKARA, Japanese). Then, SYBR green reagent (TAKARA, Japanese) was used to determine the expression of target genes during process of amplification. GAPDH was used as reference to determine loading controls, while 2^-detadeta T^ formula was used to calculate the relative expression of target genes. Primers used for the present study was shown as following: 5’- TGCGTGCTGATCGTGATCTTC-3’ (TNFSF10 forward primer), 5’- GCTCGTTGGTAAAGTACACGTA-3’ (TNFSF10 reverse primer), 5’-CTGCTCTACGACATGAACGG-3’ (ID1 forward primer), 5’-GAAGGTCCCTGATGTAGTCGAT-3’ (ID1 reverse primer), 5’-AGCTCTTCCGGGAGCCTTA-3’ (PAQR8 forward primer), 5’-GACCACCTCGTTGTGTTTCTG-3’ (PAQR8 reverse primer)5’-TCCGCCTAAGATGGGAGACC-3’ (OSR2 forward primer), 5’- GGTAAAGTGTCTGCCGCAAAA -3’ (OSR2 reverse primer), 5’- GGAGCATTTCTCGCGCTACA-3’ (PDK4 forward primer), 5’- ACAGGCAATTCTTGTCGCAAA -3’ (PDK4 reverse primer), 5’- AGAAACAGATTCGACAACTGGAG-3’ (PEX11B forward primer), 5’-TGATAGGTGAACAGCTCTTTTGG-3’ (PEX11B reverse primer), 5’- GTTCAACCGACTGGCATCCAA-3’ (TNIP1 forward primer), 5’-AGACGCACCCTCTTTGTTGC -3’ (TNIP1 reverse primer), 5’-CCGTGCAGTACAAGCTCTCC-3’ (FFAR2 forward primer), 5’- CTGCTCAGTCGTGTTCAAGTATT-3’ (FFAR2 reverse primer), 5’-TCCAAGTGCCGAAAAAGGAAG-3’ (JUN forward primer), and 5’- CGAGTTCTGAGCTTTCAAGGT-3’ (JUN reverse primer).

### 2.9 Statistical analysis

Statistical analysis was performed using SPSS (V. 27.0; ICM Corp., Armonk, NY, USA.) and R software (V. 3.6.2). One-way analysis of variance combined with Bonferroni test was used to determine the differences of genes in multi-groups, a *P*-value less than 0.05 was considered statistically significant. ROC curves were calculated to evaluate the reliability of the diagnostic models, while area under the curve (AUC) more than 0.7 and a P-value less than 0.05 were considered significance.

## 3 Results

### 3.1 Data preprocessing and identification of DEGs

**Figure 1:**
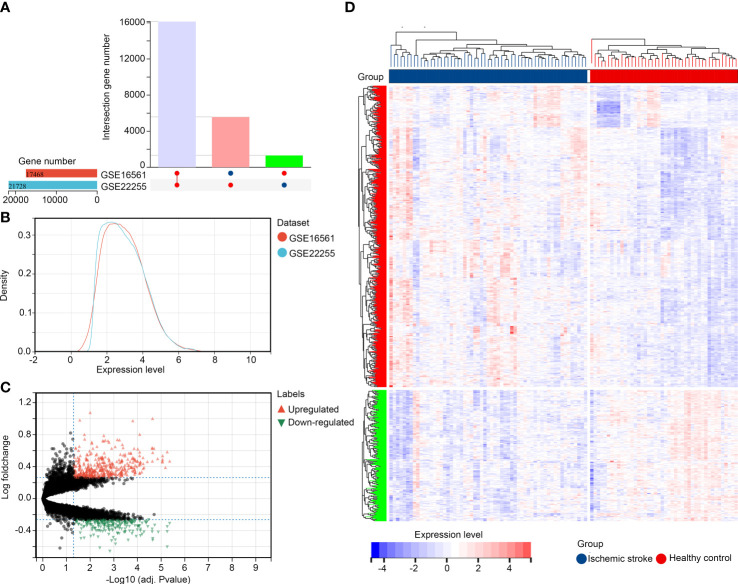
Identification of DEGs in IS **(A, B)** Merged and normalized gene expression profiles of GSE22255 and GSE16561 datasets; **(C, D)** Identification of DEGs between healthy controls and patients with IS.

Following standardization of the data formats, addition of missing values, and removal of outliers, normalized gene expression profiles of the GSE22255 and GSE16561 datasets were generated. Then, after data merging and eliminating interbatch differences between the datasets, the combined expression matrix, including 39196 gene symbols, was obtained from the samples from 59 patients with IS and 44 healthy controls in the training set (**Figures 1A, B**). Then, we performed DEG analysis to explore DEGs between these groups. A total of 579 up-regulated genes and 252 downregulated genes (**Supplementary Table 2**) were identified (**Figures 1C, D**).

### 3.2 Exploration of gene modules associated with inflammation by WGCNA

**Figure 2:**
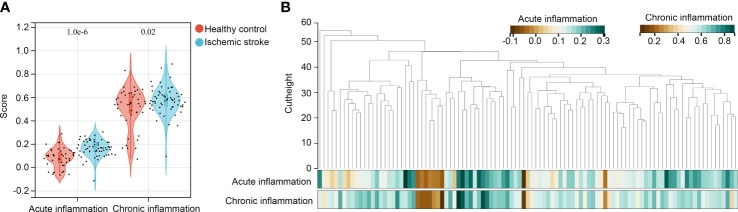
Identification of modules associated with acute and chronic inflammation scores **(A)** ssGSEA was performed to determine acute and chronic inflammation scores in patients with IS and healthy controls; **(B)** WGCNA was performed to identify co-expressed gene modules in the gene expression data of peripheral blood specimens from patients with IS and healthy controls.

We analyzed acute and chronic inflammation scores in healthy controls and patients with IS using ssGSEA. It was demonstrated that both acute and chronic inflammation scores were higher in the IS samples (**Figure 2A**). WGCNA was then performed to determine whether gene modules can simultaneously associate with the acute and chronic inflammation scores. Through preliminary estimates, we found that there were no outliers with cutheight > 60, and all samples were suitable for performing WGCNA (**Figure 2B**). In WGCNA, the soft threshold (β score) was set at 6, which can meet the scale-free topology ≥ 0.85 (**Supplement Figure 1A**), and mean connectivity was close to zero (**Supplement Figure 1B)**. Therefore, a total of 15 co-expression gene modules including black, blue, dark green, dark red, green, green-yellow, grey 60, light cyan, light green, light yellow, magenta, midnight blue, royal blue, tan, and yellow were obtained, while genes without co-expression relationships were all clustered into grey modules (**Supplement Figure 1C**). In these modules, there was the lowest adjacency in blue–light green and magenta–royal blue module pairs (**Supplement Figure 1D**).

**Figure 3:**
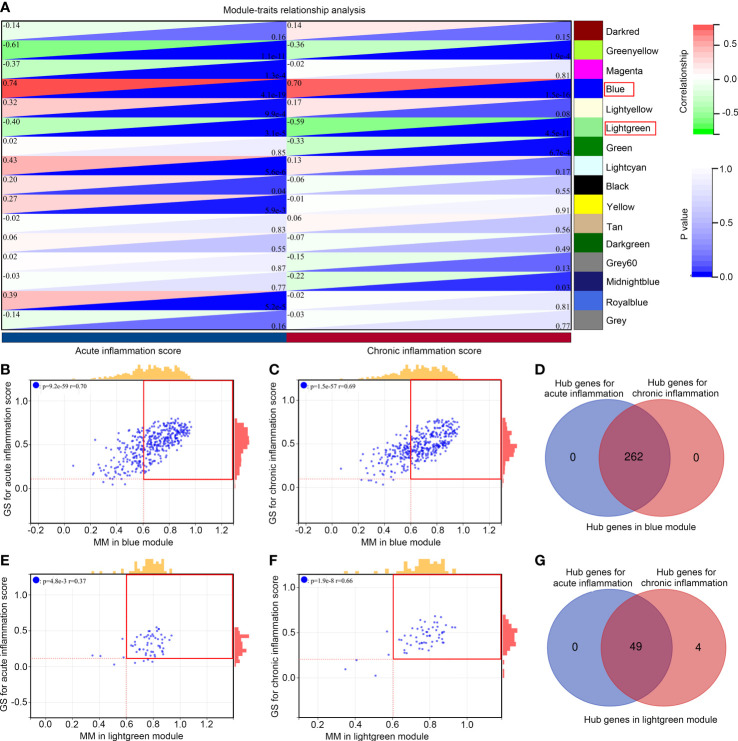
Module relationships with clinical traits **(A)** Identification of significant modules associated with clinical traits; the genes of the blue and light green modules were significantly correlated with acute and chronic inflammation scores; the relationship between gene significance (GS) and module membership (MM) in the blue **(B, C)** and light green modules **(E, F)**; Venn diagram of the hub gene intersection analysis between acute and chronic inflammation in patients with IS of the blue **(D)** and light green modules **(G)**.

Moreover, we analyzed the relationship between gene modules and inflammation scores. We found that the blue module was simultaneously and positively associated with acute (R = 0.74, P < 0.001) and chronic inflammation scores (R = 0.70, P < 0.001; **Figure 3A**), while the light green module was simultaneously and negatively associated with acute (R = -0.40, P < 0.001) and chronic inflammation scores (R =-0.59, P < 0.001; **Figure 3A**). Among 395 genes in the blue module, 262 genes met the cut-off of MM ≥ 0.6 and gene GS ≥ 0.05 for acute inflammation scores (**Figure 3B**); similarly, 262 genes met the cut-off of MM ≥ 0.6 and gene GS ≥ 0.05 for chronic inflammation scores (**Figure 3C**). In these two parts, all of the 262 genes overlapped, and these 262 genes (**Supplementary Table 3**) were set as hub genes in the blue module (**Figure 3D**). Furthermore, among 58 genes in the light green module, 49 met the cut-off of MM ≥ 0.6 and gene GS ≥ 0.05 for acute inflammation scores (**Figure 3E**); similarly, 53 genes met the cut-off of MM ≥ 0.6 and gene GS ≥ 0.05 for chronic inflammation scores (**Figure 3F**). In these two parts, the 49 genes overlapped, and these 49 genes (**Supplementary Table 4**) were set as hub genes in the light green module (**Figure 3G**). These 311 hub genes in significant modules associated with acute and chronic inflammation scores were used for further study.

### 3.2 Enrichment analysis for hub genes that were differentially expressed between IS and healthy controls

**Figure 4:**
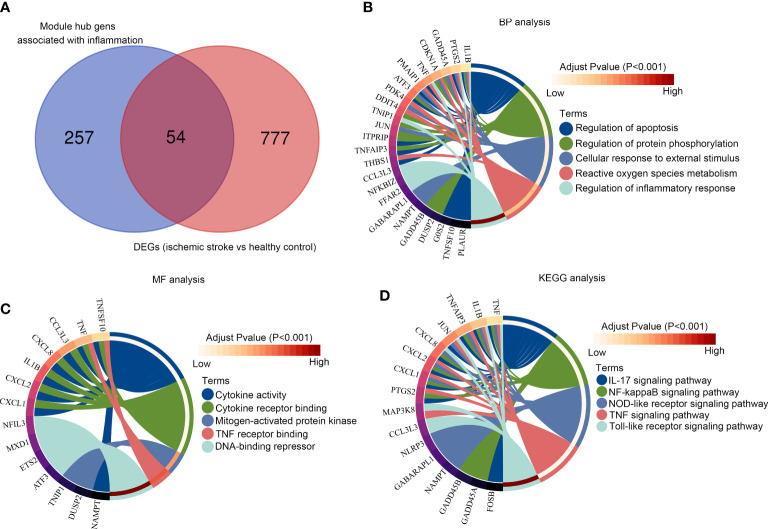
Functional enrichment analysis of the module core genes **(A)** Venn diagram of the hub gene intersection analysis between acute/chronic inflammation and DEGs in IS; **(B)** The intersection hub genes enriched in BPs; **(C)** The intersection hub genes enriched in MF; **(D)** Kyoto Encyclopedia of Genes and Genomes pathway enrichment analysis of the intersection hub genes.

Among the 311 hub genes in significant modules, 54 of them were also DEGs between patients with IS and healthy controls (**Figure 4A**). Through biological process enrichment analysis, we found that these genes were enriched in “regulation of apoptosis,” “regulation of protein phosphorylation,” “cellular response to external stimulus,” “reactive oxygen species metabolism,” and “regulation of inflammation response” (**Figure 4B**). For molecular function enrichment analysis, these 54 genes were enriched in “cytokine activity,” “cytokine receptor binding,” “mitogen-activated protein kinase,” “TNF receptor binding,” and “DNA binding repressor” (**Figure 4C**). Moreover, we found that these 54 genes were enriched in the pathways including the “IL-17 signaling pathway,” “NF-kappaB signaling pathway,” “NOD-like receptor signaling pathway,” “TNF signaling pathway,” and “Toll-like receptor signaling pathway” (**Figure 4D**).

### 3.3 Selection of core genes and construction of a diagnostic model for IS

**Figure 5:**
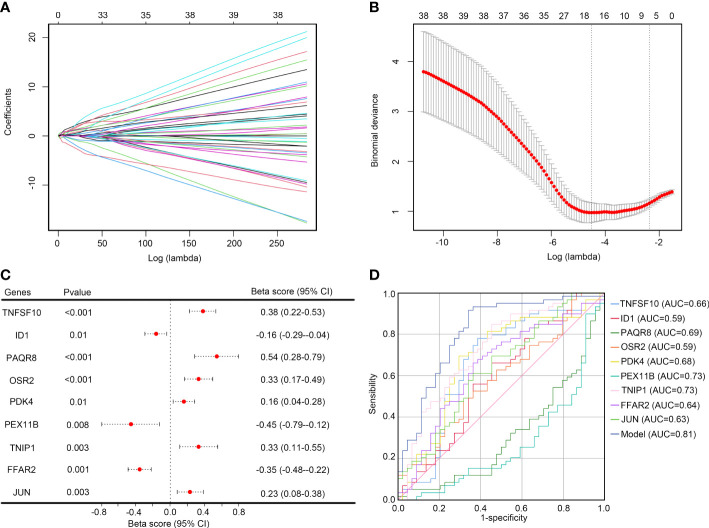
Selecting the optimal key inflammation-related genes to construct the final diagnostic model **(A)** Screening of the optimal parameters and the vertical lines were drawn; **(B)** LASSO coefficient profiles of the 17 key inflammation-related genes; **(C)** Multivariate logistic regression determined independent candidate diagnostic biomarkers; **(D)** ROC analysis showing that this diagnostic model had good diagnostic performance.

LASSO analysis was performed on the 54 key inflammation-related genes. After removing the collinearity genes, 17 genes including *ZFP3*, *TNFSF10*, *SLC2A3*, *ID1*, *PAQR8*, *OSR2*, *TNF*, *SGK1*, *GABARAPL1*, *TNFAIP3*, *PDK4*, *PEX11B*, *TNIP1*, *FFAR2*, *MXD1*, *JUN*, and *UBR4* were retained (**Figures 5A, B**). Then, multivariate LASSO regression analysis was performed, and 9 genes were selected as hub genes for IS, including *TNFSF10*, *ID1*, *PAQR8*, *OSR2*, *PDK4*, *PEX11B*, *TNIP1*, *FFAR2*, and *JUN* (**Figure 5C**). These genes were then used to construct a diagnostic model based on their expression and tolerance limits. The diagnostic model was 0.29 × *TNFSF10* + 0.45 × *ID1* - 0.207 × *PAQR8* + 0.268 × *OSR2* + 0.332 × *PDK4* - 0.233 × *PEX11B* + 0.425 × *TNIP1* + 0.235 × *FFAR2* + 0.234 × *JUN*. Through ROC analysis, we found that the diagnostic value of this model (AUC = 0.81) was significantly higher than for each single gene (**Figure 5D**).

### 3.4 Validation of gene expression and diagnostic model performance in whole blood samples

**Figure 6:**
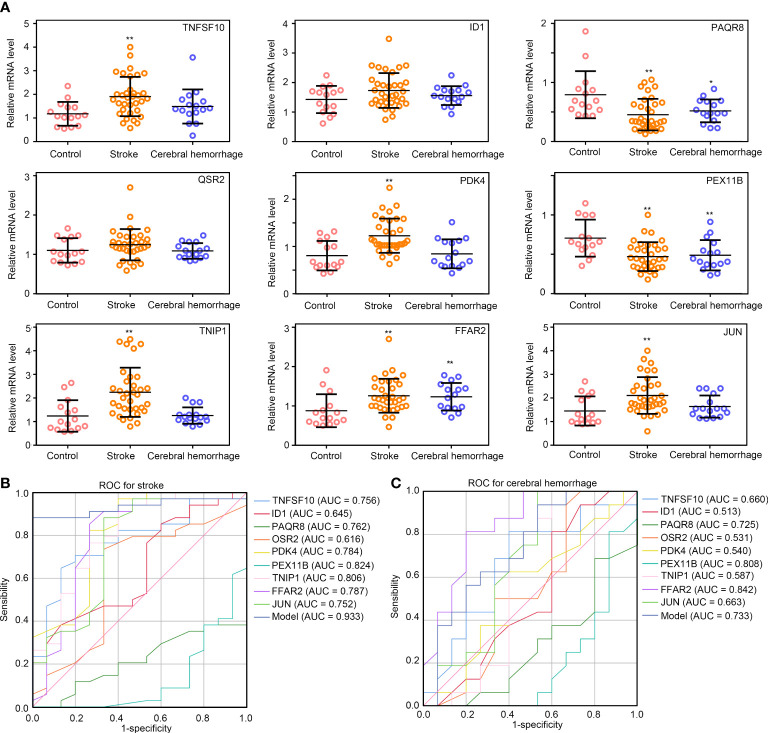
Validation of the diagnostic model in IS and CH samples. **(A)** The relative expression levels of TNFSF10, ID1, PAQR8, OSR2, PDK4, PEX11B, TNIP1, FFAR2, and, JUN in healthy controls, patients with IS and, patients with CH; **(B, C)** ROC curve analysis of TNFSF10, ID1, PAQR8, OSR2, PDK4, PEX11B, TNIP1, FFAR2, JUN, and diagnostic model constructed by these combines gene expression levels in IS and CH. **P<0.01; *P<0.05.

To verify the effectiveness of diagnostic model, we collected whole blood from healthy controls (n =15), patients with IS (n = 34), and patients with CH (n = 16). For IS, 6 patients had large-artery atherosclerotic stroke (LAA), 4 had cardiac cerebral embolism (CE), 10 had small arterial lacunar stroke (SAA), 11 had stroke of other undemonstrated etiology (SUE), and 3 had stroke from other causes (SOE) (**Supplementary Figure 2**). As shown in **Figure 6A**, the expression levels of TNFSF10, PDK4, TNIP1, FFAR2, and JUN were significantly higher in the patients with IS compared to healthy controls, and the expression levels of PAQR8 and PEX11B were lower (p < 0.01). However, the expression levels of ID1 and QSR2 were not significantly different between the patients with IS and healthy controls. Moreover, reduced expression of PAQR8 and PEX11B and elevated expression of FFAR2 were observed in patients with CH compared to healthy controls (p < 0.05). However, there was no difference in expression of TNFSF10, ID1, PAQR8, OSR2, PDK4, TNIP1, FFAR2, and JUN between subtypes of IS (LAA, CE, SAA, SUE and SOE) (**Supplementary Figure 2**). Only the expression of PEX11B was lower in CE, SUE, and SOE compared with LAA and SAA (**Supplementary Figure 2**). These results may indicate that TNFSF10, PDK4, TNIP1, and JUN may be real and specific biomarkers for IS, while PAQR8, PEX11B, and FFAR2 may be universal biomarkers for brain diseases with inflammation.

Furthermore, ROC analysis was performed, and the AUC values of TNFSF10, ID1, PAQR8, OSR2, PDK4, PEX11B, TNIP1, FFAR2, and JUN for stroke were 0.756, 0.645, 0.762, 0.616, 0.784, 0.824, 0.806, 0.787, 0.752, and 0.752, respectively, in validation samples (**Figure 6B**). We then plugged the gene expressions of TNFSF10, ID1, PAQR8, OSR2, PDK4, PEX11B, TNIP1, FFAR2, and JUN into the formula of the diagnostic model. Interestingly, the diagnostic model using these combines gene expression levels exhibited extremely high diagnostic value for IS (AUC = 0.933) and was superior to using any single gene (**Figure 6B**). Even though the combined diagnostic model exhibited a certain diagnostic value for CH (AUC = 0.733), however, it was not superior to using any single gene (**Figure 6C**). These results may indicate that the diagnostic model constructed with TNFSF10, ID1, PAQR8, OSR2, PDK4, PEX11B, TNIP1, FFAR2, and JUN expression data can be a useful tool for the diagnosis of IS.

### 3.5 The diagnostic score can reflect the condition of immune-related cells in patients with IS

**Figure 7:**
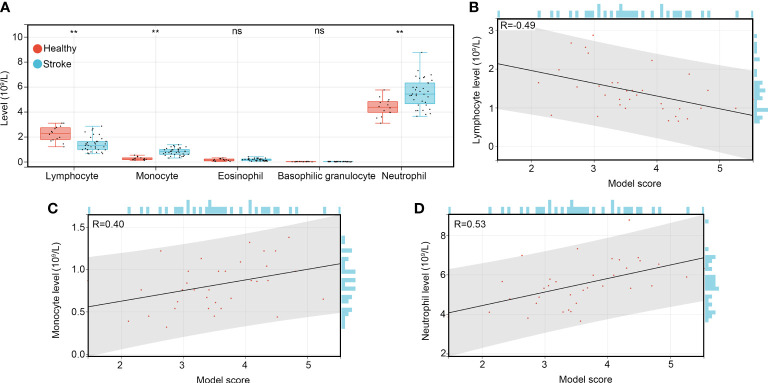
The diagnostic model can reflect the condition of immune-related cells in patients with IS. **(A)** The levels of immune-related cells such as lymphocytes, monocytes, eosinophils, basophilic granulocytes and neutrophils in the whole blood of healthy controls, patients with IS, and patients with CH. **(B)** The co-expression relationship between the diagnostic model score and levels of lymphocytes. **(C)** The co-expression relationship between the diagnostic model score and levels of monocytes. **(D)** The co-expression relationship between the diagnostic model score and levels of neutrophils. **P<0.01; ns, no significance, P>0.05.

In order to determine whether the diagnostic model score can reflect the condition of immune-related cells in patients with IS. We reviewed the results of blood routine tests of healthy controls and patients with IS, we found that the levels of lymphocytes were decreased in patients with IS compared to healthy controls, while the levels of monocytes and neutrophils were increased (**Figure 7A**). Interestingly, we found that the score calculated by the diagnostic model was negatively associated with the levels of lymphocytes (**Figure 7B**), while it was positively associated with the levels of monocytes (**Figure 7C**) and neutrophils (**Figure 7D**). These results suggested that the diagnostic model score can reflect the condition of the immune-related cells in patients with IS.

## 4 Discussion

IS affects millions of people annually across the world (20). Survivors of stroke often struggle to live independently, and they are more likely to develop additional neurological sequelae, such as dementia (21), which causes a heavy burden on patients’ families and society as a whole (20). Investigators have realized that further understanding of the pathological mechanisms of IS can reveal valuable blood biomarkers for rapid and early diagnosis and widens the time window for thrombolytic therapy. Currently, however, there is little research on whether genes and proteins involved in inflammation could serve as diagnostic biomarkers of IS.

There is evidence that IS and other acute brain diseases are characterized by an inflammatory reaction in brain tissue (22). As a result of this inflammation, multiple cytokines are released in both damaged cerebral tissue and peripheral blood (23). In the current work, each sample from the GSE16561 and GSE22255 datasets were scored based on the genes associated with acute/chronic inflammation using the ssGSEA algorithm and showed higher acute/chronic inflammation scores in patients with IS than in healthy controls; inflammatory responses may contribute to the pathological processes of IS. In addition, gene enrichment analysis indicated that these key genes from the interaction analysis were mainly involved in inflammatory or immune-related signaling pathways. Upon further analysis, nine inflammation-related genes (TNFSF10, ID1, PAQR8, OSR2, PDK4, PEX11B, TNIP1, FFAR2, and JUN) were selected to construct a diagnostic model, and the model exhibited remarkable diagnostic value for IS with an AUC of 0.81.

TNFSF10, also called TNF-related apoptosis-inducing ligand (TRAIL), is a member of the tumor necrosis factor (TNF) ligand family (24). TRAIL indeed plays a role in the regulation of innate and adaptive immunity, making it a highly intriguing molecule for several immunological disorders (25), including IS (26). Earlier studies have shown that an increased level of TRAIL on the surface of CD4^+^ T cells was strongly correlated with plaque instability in carotid atheroma tissues (27); TRAIL exerted pleiotropic activation effects on endothelial cells, vascular smooth muscle cells, and inflammation cells (28); and low levels of TRAIL were linked to a poor prognosis in individuals with acute myocardial infarction, according to multiple clinical trials (29). A recently published study revealed that low serum TRAIL levels were associated with acute IS severity (30), while its diagnostic value was not assessed. Pyruvate dehydrogenase kinase 4 (PDK4), a member of the PDK family, regulates pyruvate dehydrogenase complexes in the CNS, which have important effects on neuron–glia metabolic interactions (31). A recent bioinformatic study identified it as an autophagy-related gene and diagnostic marker of major depressive disorder with an AUC of 0.62 (32). TNFα-induced protein 3-interacting protein 1 (TNIP1), is increasingly being recognized as a key blocker of inflammatory signaling, and its dysfunction or deficiency may predispose healthy cells to inflammatory responses (33). Some researchers have shown that anti-inflammatory therapeutic targets based on TNIP1 may be developed and tested in the future (33). Free fatty acid receptor 2 (FFAR2) is involved in immune responses and is expressed in white blood cells (34). We identified TNIP1 and FFAR2 as inflammation-related genes in the current work, and these could serve as diagnostic biomarkers in IS, although there are no studies on the relationships between TINP1/FFAR2 and IS. As one of the most extensively studied proteins of the activator protein-1 (AP-1) complex, c-JUN is involved in a multitude of cellular functions, including proliferation, apoptosis, survival, tumorigenesis, and tissue morphogenesis (35). Increasing evidence has been shown for the interaction of Notch with NF-κB, HIF-1α, JNK/c-JUN, Pin1, and p53 in stroke, while their specific regulatory mechanisms have not been elucidated (36). In the future, understanding the relationship between JUN and IS from the perspective of the inflammatory response may be a new research direction. In our own validation cohort, five of the abovementioned genes (TNFSF10, PDK4, TNIP1, FFAR2, and JUN) were also found to be highly expressed and of high diagnostic value in patients with IS, with AUCs all greater than 0.7. In particular, the FFAR2 expression level was also increased in patients with CH and had good diagnostic value (AUC = 0.842).

As a member of the progestin and adipoQ receptor (PAQR) family, PAQR8 regulates a wide range of cognitive, neuroendocrine, neuroimmune, and neuroprotective functions (37). One study suggested that baicalin and/or jasminoidin alleviated cerebral ischemia through upregulating PAQR8 expression in the rat hippocampus (38). Peroxisome membrane protein 11B (PEX11B) participates in the proliferation and division of the peroxisome itself, and the peroxisome is an organelle that contains a variety of enzymes that scavenge reactive oxygen species (39). It has been shown that a rapid increase in reactive oxygen species production after acute IS rapidly overwhelms antioxidant defenses, causing further tissue damage (40). Consistent with what we found in our validation cohort, PAQR8 and PEX11B were downregulated in both patients with IS and those with CH. This suggests that, on the one hand, inflammation is one of the broad-spectrum drivers in multiple brain injury diseases, and, on the other hand, a single gene as a biomarker has the limitation of low specificity.

ID1, named DNA binding inhibitor 1, is highly expressed in the central nervous system (CNS) during embryogenesis and throughout adulthood, and it may play a role in the molecular mechanisms regulating the cellular responses to TNFα and CNS inflammation (41). OSR2, odd-skipped related transcription factor 2, plays a critical role in cellular proliferation and quiescence under epigenetic regulation (42). Additionally, Ma et al. identified OSR2 as an immune infiltration-associated gene in sciatica with high diagnostic value (43). However, the relationship between ID1 and IS has not been reported in the literature. Unfortunately, there was also no significant change in the expression level of ID1/OSR2 in our validation cohort compared with healthy controls, and further studies in a larger sample are needed.

Interestingly, we found that the current diagnostic models had a higher diagnostic value than using single genes. In addition, the diagnostic model constructed based on IS had better diagnostic performance than that based on CH (AUC_IS_ = 0.933, AUC_CH_ = 0.733), suggesting that the novel diagnostic model constructed using inflammation-related genes had a better diagnostic specificity. Although some single-gene biomarkers have shown excellent diagnostic value, they are not good at distinguishing disease traits. IS is a complex (multifactorial) polygenic disease that results from the interaction of risk factors and genetic components caused by polymorphic genes acting independently or fueling each other. Therefore, these data can be combined in multivariate analyses in the future, using factors such as oxidative stress and endothelial activation, to determine the best diagnostic markers for IS and optimize the diagnostic value of single genes.

Increasing evidence indicates that peripheral immune-inflammatory pathways are activated following stroke, which play a critical role in neurological outcomes (44). Studies on immune responses following stroke have revealed that CD4^+^CD25^+^Foxp3^+^ regulatory T cells play crucial and complex roles in controlling the inflammatory damage caused by stroke as well as modulating immunosuppression (45). For instance, in a rat model of stroke, the depletion of T-lymphocytes led to smaller volumes of cerebral infarction and better recovery of neural function compared to controls (46); in patients with acute IS, T cells are activated in the peripheral blood (47, 48). Monocytes in the blood may mediate neuroinflammatory responses and strongly influence IS outcomes (49, 50). We found that lymphocyte levels were lower in patients with IS compared with healthy controls, while the levels of monocytes and neutrophils were higher. In addition, this diagnostic model could reflect the main immune-related cell status of IS patients to a certain extent, suggesting that we can understand disease status using this diagnostic model.

We should acknowledge that there are some limitations to the present work. First, the data we analyzed were from public databases and the sample size was small, although we integrated two datasets. Second, although the AUC of the model exhibited acceptable diagnostic ability, the model performance needs to be improved. For example, the current diagnostic model cannot provide relevant information on the severity and subtype of IS. Because of the limited data we collected, some biomarkers were not ideal for the subtype analysis. We could only demonstrate that these biomarkers had good specificity in the diagnosis of IS. However, for the subtype analysis, a larger sample is still needed for further analysis. Finally, the current diagnostic model targets only inflammation-related genes, but similar in-silico studies of oxidative stress and endothelial activation are needed to develop optimal diagnostic markers for IS.

## 5 Conclusions

In summary, the diagnostic model constructed by the inflammation-related genes TNFSF10, ID1, PAQR8, OSR2, PDK4, PEX11B, TNIP1, FFAR2 and JUN exhibited high and specific diagnostic value for IS, and reflected the condition of lymphocytes, monocytes and neutrophils in blood. The novel diagnostic model may contribute to the diagnosis of IS.

## Data availability statement

The original contributions presented in the study are included in the article/**Supplementary Material**. Further inquiries can be directed to the corresponding authors.

## Ethics statement

The studies involving human participants were reviewed and approved by the Ethics Committee of Biomedicine in Seventh Medical Center of Chinese PLA General Hospital, Beijing, China. (Approval number: 2022-182). Written informed consent for participation was not required for this study in accordance with the national legislation and the institutional requirements.

## Author contributions

PR, J-YW, and Z-RZ analyzed the data, prepared figures, and wrote the manuscript. X-WL and H-LC helped in analyzing the data and visualization. Y-FL, W-ZG, and Y-QZ conceived and designed the study. All authors contributed to the article and approved the submitted version.

## Supplementary material

Supplementary Figure 1 Constructing the WGCNA **(A)** Scale independence of various soft-threshold values; **(B)** Mean connectivity of various soft-threshold values; **(C)** Clustering dendrograms of all genes with dissimilarity based on the topological overlap, together with assigned module colors; **(D)** The distance in 16 gene co-expression modules

Supplementary Figure 2 The levels of TNFSF10, ID1, PAQR8, OSR2, PDK4, PEX11B, TNIP1, FFAR2, and JUN in each subtype of IS including LAA, CE, SAA, SUE and SOE.
