# Supplementary figures and images for: Diagnostic model constructed by nine inflammation-related genes for diagnosing ischemic stroke and reflecting the condition of immune-related cells
Source: Front Immunol. 2022 Dec 13;13:1046966. doi: 10.3389/fimmu.2022.1046966 (PMC9792959; doi:10.3389/fimmu.2022.1046966)

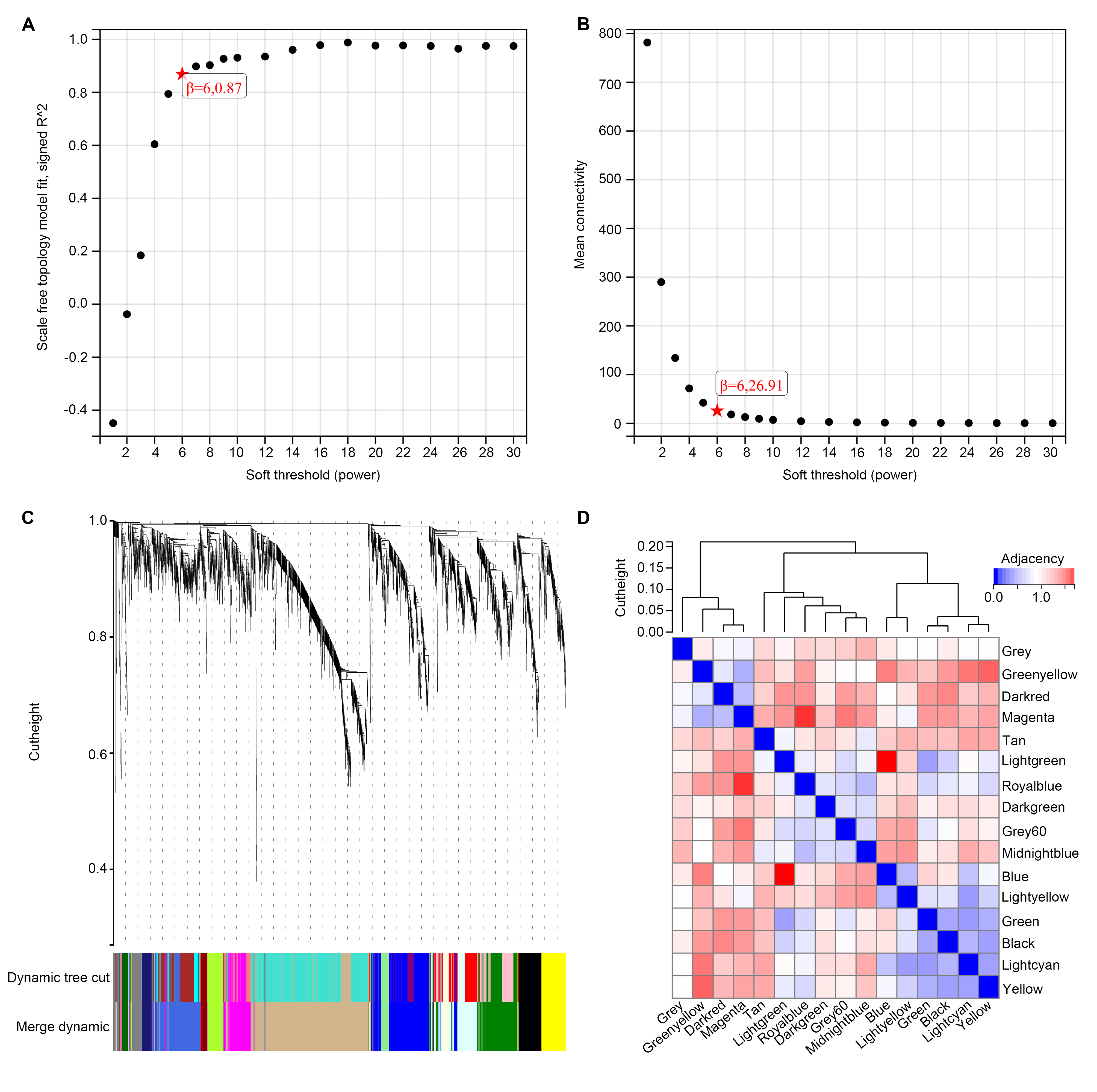

Supplement: Supplementary Figure 1 — Constructing the WGCNA (A) Scale independence of various soft-threshold values; (B) Mean connectivity of various soft-threshold values; (C) Clustering dendrograms of all genes with dissimilarity based on the topological overlap, together with assigned module colors; (D) The distance in 16 gene co-expression modules [file Image_1.tif]

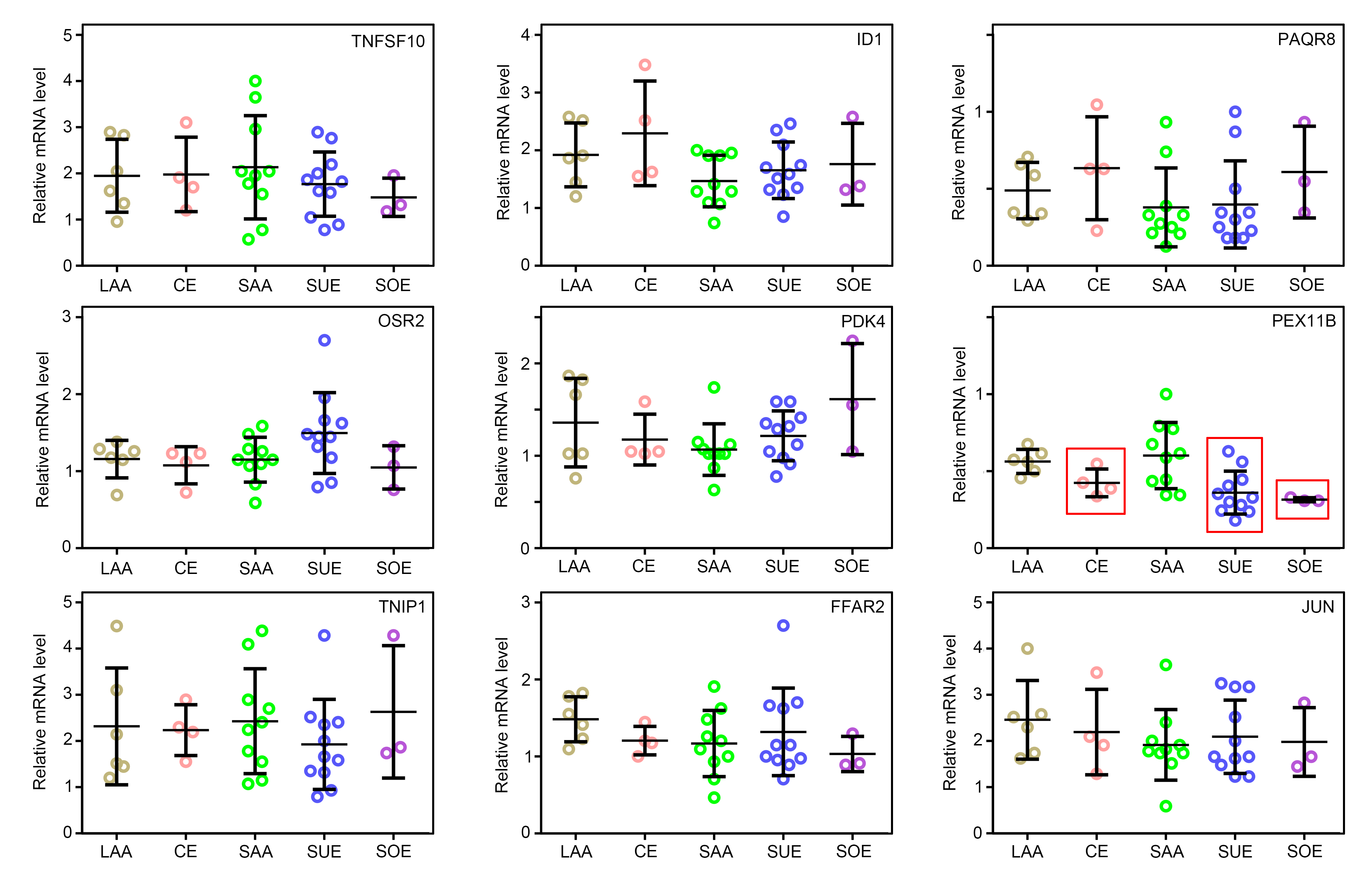

Supplement: Supplementary Figure 2 — The levels of TNFSF10, ID1, PAQR8, OSR2, PDK4, PEX11B, TNIP1, FFAR2, and JUN in each subtype of IS including LAA, CE, SAA, SUE and SOE. [file Image_2.tif]
